# *Burkholderia cenocepacia* Type VI Secretion System Mediates Escape of Type II Secreted Proteins into the Cytoplasm of Infected Macrophages

**DOI:** 10.1371/journal.pone.0041726

**Published:** 2012-07-25

**Authors:** Roberto Rosales-Reyes, Daniel F. Aubert, Jennifer S. Tolman, Amal O. Amer, Miguel A. Valvano

**Affiliations:** 1 Centre for Human Immunology, Department of Microbiology and Immunology, The University of Western Ontario, London, Ontario, Canada; 2 Laboratorio de Infectología, Microbiología e Inmunología Clínicas, Departamento de Medicina Experimental, Facultad de Medicina, Universidad Nacional Autónoma de México, Mexico City, México; 3 Centre for Microbial Interface Biology, Department of Microbial Infection and Immunity and the Department of Internal Medicine, Ohio State University, Columbus, Ohio, United States of America; National Jewish Health and University of Colorado School of Medicine, United States of America

## Abstract

*Burkholderia cenocepacia* is an opportunistic pathogen that survives intracellularly in macrophages and causes serious respiratory infections in patients with cystic fibrosis. We have previously shown that bacterial survival occurs in bacteria-containing membrane vacuoles (BcCVs) resembling arrested autophagosomes. Intracellular bacteria stimulate IL-1β secretion in a caspase-1-dependent manner and induce dramatic changes to the actin cytoskeleton and the assembly of the NADPH oxidase complex onto the BcCV membrane. A Type 6 secretion system (T6SS) is required for these phenotypes but surprisingly it is not required for the maturation arrest of the BcCV. Here, we show that macrophages infected with *B. cenocepacia* employ the NLRP3 inflammasome to induce IL-1β secretion and pyroptosis. Moreover, IL-1β secretion by *B. cenocepacia-*infected macrophages is suppressed in deletion mutants unable to produce functional Type VI, Type IV, and Type 2 secretion systems (SS). We provide evidence that the T6SS mediates the disruption of the BcCV membrane, which allows the escape of proteins secreted by the T2SS into the macrophage cytoplasm. This was demonstrated by the activity of fusion derivatives of the T2SS-secreted metalloproteases ZmpA and ZmpB with adenylcyclase. Supporting this notion, ZmpA and ZmpB are required for efficient IL-1β secretion in a T6SS dependent manner. ZmpA and ZmpB are also required for the maturation arrest of the BcCVs and bacterial intra-macrophage survival in a T6SS-independent fashion. Our results uncover a novel mechanism for inflammasome activation that involves cooperation between two bacterial secretory pathways, and an unanticipated role for T2SS-secreted proteins in intracellular bacterial survival.

## Introduction

*Burkholderia cenocepacia* belongs to the *Burkholderia cepacia* complex (Bcc) [1], a group of opportunistic bacteria that cause respiratory tract infections in patients with cystic fibrosis (CF) [2]. *B. cenocepacia* infection can lead to rapid decline of lung function and in some cases to fatal “cepacia syndrome” [3]. The virulence of *B. cenocepacia* and other Bcc bacteria is multifactorial [4], and relies in part on their ability to survive within a membrane-bound vacuole in amoebae, human respiratory epithelial cells and macrophages [5]. In macrophages, the maturation of the *B. cenocepacia*-containing vacuole (BcCV) is delayed, as revealed by impaired acidification and fusion with lysosomes [6], Rab7 inactivation [7], and Rac1 inactivation [8], [9]. Also, the intracellular infection by *B. cenocepacia* causes downregulation of the autophagy pathway [10].

*B. cenocepacia* infections are persistent and characterized by exacerbated inflammatory responses [11]–[14]. Intracellular bacterial survival may explain the persistence of the infection and also contribute to proinflammatory responses that become detrimental to the patient [5]. Initiation of the inflammatory response upon bacterial infection requires participation of cell membrane and cytoplasmic recognition systems. The Toll-like receptors (TLRs) usually recognize pathogen-associated molecular patterns (PAMPs) directly at the plasma or vacuolar membranes, leading to the synthesis and secretion of specific cytokines, including pro-IL-1β and pro-IL-18 [15]. This mechanism operates in macrophages infected by *B. cenocepacia* whereby the bacterial O antigen lipopolysaccharide contributes to TLR4-mediated IL-1β secretion [16]. PAMP recognition in the cytoplasm depends on a family of nucleotide-binding oligomerization domain-like receptor (NLR) proteins that mediate caspase-1 activation, which in turns regulates the proteolytic processing of inactive precursors pro-IL-1β and pro-IL-18 into mature IL-1β and IL-18 respectively and their subsequent release [17].

Pathogen recognition and caspase-1 activation can trigger pyroptosis [18], an inherently inflammatory, programmed cell death that differs from apoptosis and necrosis [19]. The NLR family, including NLRP1, NLRP3, NLRC4 and the apoptosis-associated speck-like protein containing a caspase recruitment domain (ASC) adaptor, are critical components of the inflammasome linking microbial and endogenous ‘danger’ signals to caspase-1 activation and IL-1β secretion [17]. Upon engulfment, pathogens, crystals and other substances can damage the phagolysosomal membrane leading to subsequent release of endogenous molecules such as cathepsin-B, which is detected by NLRP3 and triggers the activation of the NLRP3 inflammasome [20]. ASC is an adaptor molecule that mediates inflammatory and apoptotic signals [17] linking intracellular NLR proteins such as NLRP3 to caspase-1 through direct physical association [21].

Bacterial intracellular survival typically relies on one or more specialized secretory systems that inject bacterial molecules into the cytoplasm of eukaryotic cells. These molecules often affect host-signaling pathways and allow bacteria to establish an intracellular infection [22], [23]. The *B. cenocepacia* Type VI secretion system (T6SS) affects the actin cytoskeleton of infected macrophage [24] and the NADPH oxidase complex assembly at the BcCV by an as yet unknown mechanism that results in the inactivation of Rac1 and Cdc42 [8], [9]. The T6SS apparatus is thought to puncture the membrane of eukaryotic cells, allowing the translocation of T6SS-effector molecules into the infected cell [25]. The T2SS of *B. cenocepacia* mediates the secretion of several bacterial proteins such as ZmpA and ZmpB, which are Zn^2+^-dependent metalloproteases that degrade a wide range of substrates including innate immune mediators [26], [27]. Although T2SS effector proteins are released to the bacterial extracellular milieu they cannot be directly injected into the host cell cytosol. In this work, we reveal a novel mechanism of inflammasome activation by *B. cenocepacia* involving the combination of two secretory systems. A functional T6SS is required to mediate the translocation of T2SS-effectors from the BcCV to the cytosol, presumably by damaging the BcCV membrane; once in the host cytosol, these T2SS effectors trigger IL-1β secretion and pyroptosis via the NLRP3/ASC inflammasome.

## Results

### IL-1β Secretion by *B. cenocepacia*-infected Macrophages is Associated with Membrane Damage, Pyroptosis, and Activation of the NLRP3/ASC Inflammasome

**Figure 1:**
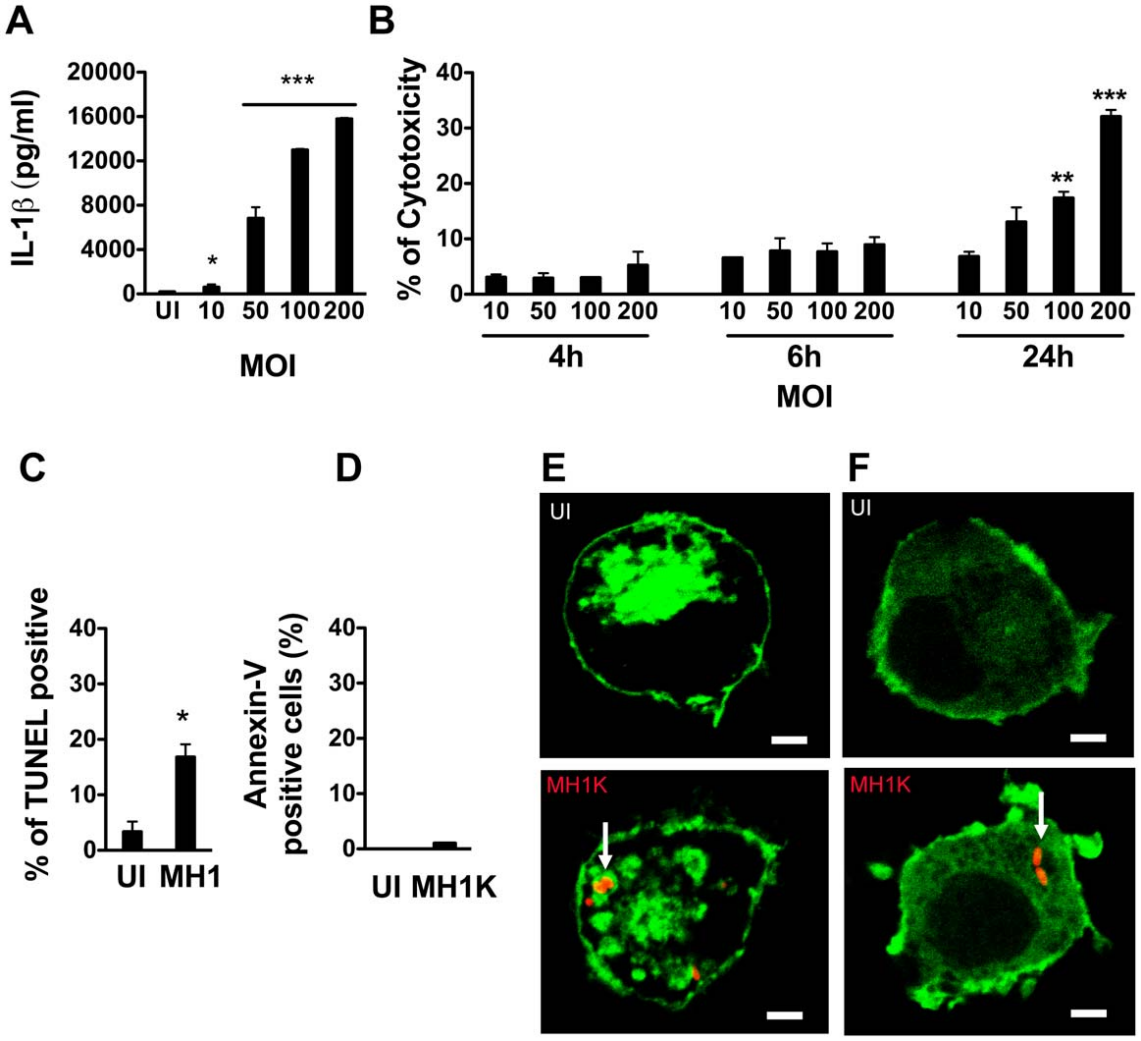
*Burkholderia cenocepacia* induces pyroptosis in macrophages. Graphs in (A) through (D) represent mean ± SEM from three independent experiments. A. Macrophages were infected with *B. cenocepacia* MH1K at different MOIs for 1 h and then treated with gentamicin as described in Experimental Procedures. ELISA was used to quantify IL-1β in cell supernatants at 24 h post-infection. * *p*≤0.05 and *** *p*≤0.001 values compared to uninfected (UI) macrophages. B. Macrophages were infected as in (A) and the supernatants were used to quantify total lactate dehydrogenase (LDH) activity at 4, 6 and 24 h post-infection. ** *p*≤0.01 and *** *p*≤0.001 values for MOI of 10 at 24 h post-infection. C and D. Macrophages were infected as in (A) and at 24 h post-infection stained with the TUNEL-AF488 kit (C) or with Annexin V-AF488 (D) * *p*≤0.05 values compared to UI macrophages. Stained cells were analyzed by flow cytometry. E and F. Confocal images at 24 h post-transfection of macrophages expressing the fluorescent probe Lact-C2-GFP (Green) (E) or PH-PLCδ-GFP (Green) (F) Upper panels show UI macrophages. Lower panels show macrophages infected with *B. cenocepacia* MH1K-RFP (Red) for 4 h. Scale bar, 10 µm. Arrows indicate BcCVs.

We have previously demonstrated that *B. cenocepacia* K56-2-infected murine macrophages produce the proinflammatory cytokine IL-1β in a TLR4- and caspase-1-dependent manner [16]. To determine whether *B. cenocepacia* induces pyroptosis, we first investigated the release of IL-1β by ANA-1 macrophages (herein macrophages) infected with the strain MH1K (Table 1). This strain is an isogenic derivative of K56-2 that carries a deletion of an efflux pump resulting in bacterial sensitivity to gentamicin, and allowing us to efficiently kill extracellular bacteria [28]. Using this strain eliminates potentially confounding effects on macrophages that remain continuously exposed to extracellular bacteria, as in the case of the gentamicin-resistant K56-2 strain. At 24 h post-infection, macrophages infected with a multiplicity of infection (MOI) of 50 produced 6 831±896 pg/ml of IL-1 β (Figure 1A), and exhibited 13±2.7% cytotoxicity measured by lactate dehydrogenase release (Figure 1B). IL-1β and cytotoxicity levels correlated with the MOI (12 989±86 pg/ml and 17.4±1.1% cytotoxicity at a MOI of 100, and 15 789±86 pg/ml and 32±1.2% cytotoxicity at an MOI of 200; Figure 1A and 1B). Cytotoxicity was associated with chromosomal DNA double-strand breaks by the TUNEL fragmentation assay (Figure 1C) and lack of exposure of phosphatidylserine at the cell surface (Figure 1D). Macrophages transfected with the fluorescent chimeric probe Lact-C2-GFP that binds to cytosolic exposed phosphatidylserine showed fluorescence associated with the inner leaflet of the plasma membrane as well as with BcCVs (Figure 1E). We also measured the generation of phosphoinositide 4,5-*bis*phosphate [PI(4,5)P_2_] at the inner leaflet of the plasma membrane by transfecting the macrophages with GFP-PLCδ-PH, a chimeric protein consisting of the PH domain of phospholipase Cδ fused to GFP. By comparison with uninfected cells (Figure 1F, top panel) *B. cenocepacia* infection did not alter the normal distribution of the GFP-PLCδ-PH probe at the plasma membrane and no GFP-PLCδ-PH signal was detectable on the BcCV (Figure 1F, bottom panel). These data, in agreement with previous observations by Flannagan et al. [8], indicate that the phospholipid composition of the plasma membrane is not altered during *B. cenocepacia* infection, ruling out apoptosis.

**Table 1:** Bacterial strains and plasmids used in this study.

Strain or Plasmid	Relevant Characteristics^a^	Source or Reference
*B. cenocepacia*		
K56-2	ET12 clone related to J2315, CF clinical Isolate, Gm^R^	BCRRC^a^
MH1K	K56-2, Δ*amrABC* (BCAL1674–1676); Gm^S^	[28]
Δ*T2SS*	Strain JST178; MH1K derivative with Δ*gspL* (BCAL3516), defective in T2SS, Gm^S^	This study
Δ*T4SS-1*	Strain JST17; K56-2 derivative carrying ΔpBCA017–59 and Δ*amrABC* (BCAL1674–1676),defective in T4SS-1, Gm^S^	This study
Δ*T4SS-2*	Strain JST39; MH1K derivative with ΔBCAM0324–0335), defective in T4SS-2, Gm^S^	This study
Δ*T3SS*	JST40; MH1K derivative with ΔBCAM2040–2057, defective in T3SS, Gm^S^	This study
Δ*fliCD*	JST114; K56-2 derivative with Δ*fliCD* and Δ*amrABC* (BCAL1674–1676), defective inflagellin, Gm^S^	This study
*T6SS+*	Strain JST143; K56-2 derivative with Δ*atsR* and Δ*amrABC* (BCAL1674–1676), defective in negative regulator of T6SS expression, Gm^S^	This study
*T6SS+-*Δ*T2SS*	Strain JST143 derivative with Δ*gspFED* (BCAL3525–3527), defective in T2SS, Gm^S^	This study
Δ*T6SS*	JST144; K56-2 derivative with Δ*atsR*, Δ*bcsJ* (*hcp*; BCAL0343), and Δ*amrABC*(BCAL1674–1676), defective in T6SS, Gm^S^	This study
Δ*T3SS-*Δ*fliCD*	JST188; JST114 derivative with ΔBCAM2045–2057, defective in flagellin and T3SS, Gm^S^	This study
Δ*T2SS-*Δ*T6SS*	JST198; MH1K derivative with Δ*gspL* (BCAL3516) and Δ*bcsJ* (*hcp*; BCAL0343),defective in T2SS and T6SS, Gm^S^	This study
Δ*T2SS-*Δ*T6SS*	JST144 derivative with Δ*gspFED* (BCAL3525–3527), also defective in T2SS, Gm^S^	This study
*T6SS*+-Δ*zmpA*	MH1K derivative with Δ*atsR* and Δ*zmpA*, defective in ZmpA production, Gm^S^	This study
*T6SS*+-Δ*zmpB*	MH1K derivative with Δ*atsR* and Δ*zmpB*, defective in ZmpB production, Gm^S^	This study
*T6SS*+-Δ*zmpAB*	MH1K derivative with Δ*atsR,* Δ*zmpA*, and Δ*zmpB*, defective in ZmpA and ZmpB production, Gm^S^	This study
Δ*T6SS-*Δ*zmpAB*	MH1K derivative; Δ*atsR*, Δ*zmpA*, Δ*zmpB*, and Δ*bcsJ* (*hcp*; BCAL0343), defective in ZmpA and ZmpBproduction and in T6SS, Gm^S^	This study
Δ*T6SS-*Δ*T2SS-*Δ*zmpAB*	MH1K derivative; Δ*atsR*, Δ*zmpA*, Δ*zmpB*, Δ*bcsJ* (*hcp*; BCAL0343), and Δ*gspFED*(BCAL3525–3527), defective in ZmpA and ZmpB production, in T6SS, and in T2SS, Gm^S^	This study
*E. coli*		
DH5α	F- φ80*lacZ* M15 *endA1 recA1 supE44 hsdR*17(r_K_- m_K_+) *deoRthi-1 nupG supE44 gyrA96 relA1 Δ*(*lacZYA-argF*)*U169,* λ^–^	Laboratory stock
SY327	*araD,* Δ*(lac pro) argE(Am) recA56 rifR nalA,* λ *pi*	[58]
Plasmids		
pDAI-SceI	*ori*pBBR1, Tet^R^, *Pdhfr, mob+*, expressing I-SceI restriction enzyme	[56]
pDSredT3	Expresses Red fluorescent protein, Cm^R^	
pGPI-SceI	*ori*R6K, ΩTp^R^, *mob*+, I-SceI restriction site	[56]
pMS107	pIC20H derivative, contains a 1.3 kb fragment encoding the adenylcyclase from*B.* pertussis, CyaA’ (aa 2–405), Amp^R^	[43]
pRK2013	*ori*_colE1,_ RK2 derivative, Kan^R^, *mob* ^+^, *tra* ^+^	[55]
pSCrhaB2	Cloning vector inducible with rhamnose, *ori*pBBR1, *rhaR rhaS* P_rhaB_ Tp^R^ *mob*+	[57]
pSCrha-cyaA’	Cloning vector, derivative of pSCrhaB2, inducible with rhamnose used to createC-terminal fusions with CyaA’	This study
pZmpA-cyaA’	*zmpA* in pSCrha-cyaA’, CyaA’ fused to the C-terminus of ZmpA	This Study
pZmpB-cyaA’	*zmpB* in pSCrha-cyaA’, CyaA’ fused to the C-terminus of ZmpB	This Study

a Amp, ampicillin; BCRRC, *B. cepacia* complex Research and Referral Repository for Canadian CF Clinics; Cm, chloramphenicol; Gm, gentamicin; Kan, kanamycin; Tp, trimethoprim;; Tet, tetracycline; T2SS, Type II secretion system; T6SS, Type VI secretion system.

**Figure 2:**
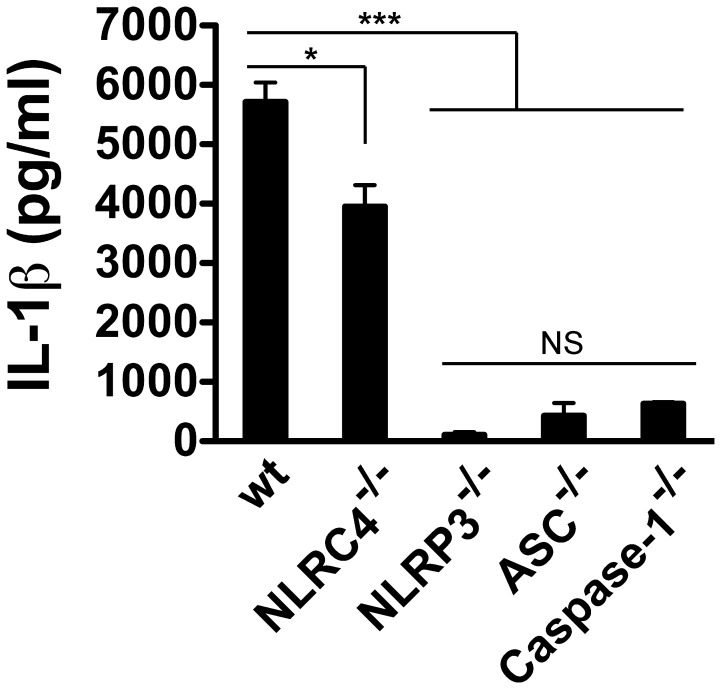
IL-1β secretion by macrophages infected with *B. cenocepacia* requires the NLRP3/ASC inflammasome. Bone marrow derived macrophages from wild type (wt), NLRC4^−/−^, NLRP3^−/−^, ASC^−/−^ and Caspase-1^−/−^ (all from C57BL/6 background) were infected with *B. cenocepacia* MH1K for 1 h at an MOI of 50 and then treated with gentamicin to eliminate extracellular bacteria. ELISA was used to quantify IL-1β in cell supernatants at 24 h post-infection. *, *p*≤0.05; ***; *p*≤0.001; NS, non significant.

To identify the NLR involved in IL-1β secretion, we infected bone marrow-derived wild type (wt) and knockout macrophages in *NLRC4*, *NLRP3*, *ASC* and *Caspase-*1 (all from C57BL/6 mice background) at MOI of 50 and quantified the IL-1β produced 24 h post-infection. The *wt* macrophages infected with *B. cenocepacia-*MH1K produced 5 714±327 pg/ml, similar to the ANA-1 macrophages (compare Figure 1A with Figure 2), while a small reduction in IL-1β secretion was detected in supernatants of *NLRC4* ^−/−^ macrophages (3 950±363 pg/ml; Figure 2). In contrast, *NLRP3* ^−/−^, *ASC* ^−/−^ and *Caspase1* ^−/−^ macrophages showed significantly reduced levels of secreted IL-1β (104±45, 441±214 and 632±27 pg/ml, respectively; Figure 2). These results indicate that IL-1β secretion by macrophages infected with *B. cenocepacia* is mediated by the NLRP3/ASC inflammasome, and requires caspase-1 as shown before [16].

**Figure 3:**
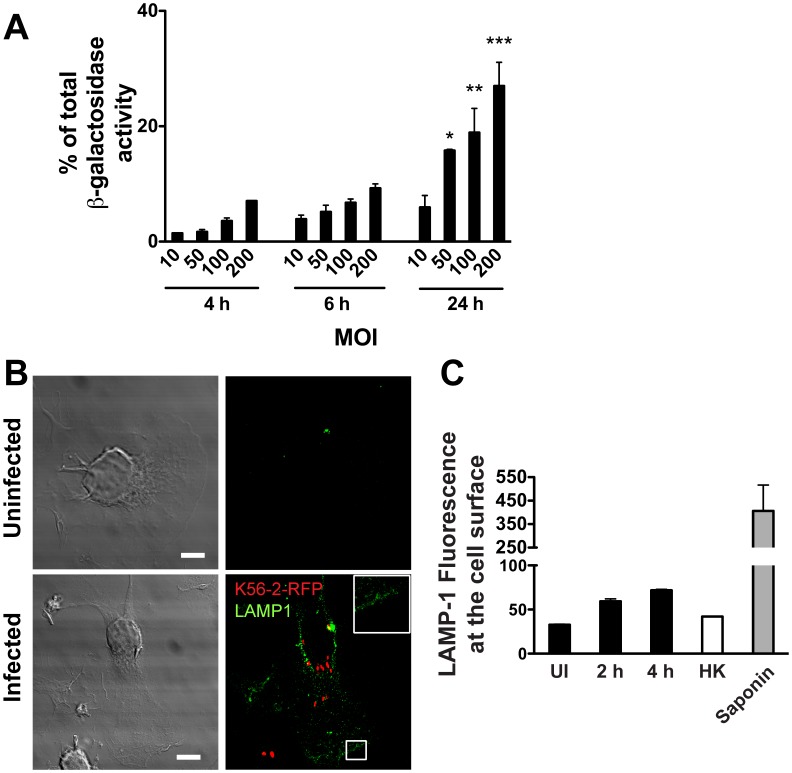
*B. cenocepacia* infection induces plasma membrane damage. Graphs represent mean ± SEM from three independent experiments. A. Macrophages were infected with *B. cenocepacia* at different MOI for 1 h and then treated with gentamicin to eliminate extracellular bacteria. The β-galactosidase activity was quantified from supernatants of infected macrophages at 4, 6 and 24 h post-infection. * *p*≤0.05; ** *p*≤0.01 and *** *p*≤0.001 values for MOI of 10 at 24 h post-infection. B. Macrophages were infected with *B. cenocepacia* K56-2-RFP (red) for 4 h. Unpermeabilized cells were fixed and stained with anti-LAMP-1 antibodies (green). Representative confocal z slice is shown. Inset is higher magnification of the boxed area. Scale bars are 10 µm. C. Macrophages were infected with *B. cenocepacia* K56-2 for 2 or 4 h. The cells were stained without permeabilization with anti-LAMP-1 antibodies (green) and analyzed by flow cytometry. The graph shows the mean intensity of fluorescence (MIF) of LAMP1 in the cell surface. UI, uninfected macrophages; HK, macrophages infected with heat-killed bacteria; saponin, macrophages permeabilized with saponin.

One possible mechanism for the release of IL-1β involves the transport of immature IL-1β to a subset of lysosomes followed by its cleavage into its active form and release to the macrophage milieu [29], [30]. Therefore, we investigated whether IL-1β secretion in *B. cenocepacia*-infected macrophages was associated with release of lysosomal content. The supernatants of infected macrophages at MOIs of 50, 100 and 200 contained 16±0.2%, 19±4.2% and 27±4.1% of total β-galactosidase activity (a marker of lysosomal content), respectively (Figure 3A). These results mimic the correlation between cytotoxicity of macrophages and bacterial load, indicating that intracellular infection by *B. cenocepacia* induces damage to the plasma membrane, which presumably activates the membrane fusion of secretory lysosomes as a repair mechanism [31]. This idea was supported by the observation that unpermeabilized infected macrophages display LAMP-1, a lysosomal membrane marker on the cell surface, as revealed by confocal microscopy and flow cytometry using anti-LAMP-1 (Figure 3B). Moreover, the amount of LAMP-1 exposed at the cell surface increased at longer times of infection (Figure 3C). These observations also agree with the concomitant reduction in the total proteolytic activity of infected macrophages (Figure S1A and S1B), which could be explained by loss of lysosomal content to the external milieu. Together, *B. cenocepacia* infection of macrophages results in IL-1β secretion, plasma membrane damage and lysosomal fusion to the plasma membrane with the resultant release of lysosomal content, and ultimately cell death by pyroptosis.

### IL-1β Secretion by Infected Macrophages Requires Cooperation between Type VI and Type II Secretion Systems

**Figure 4:**
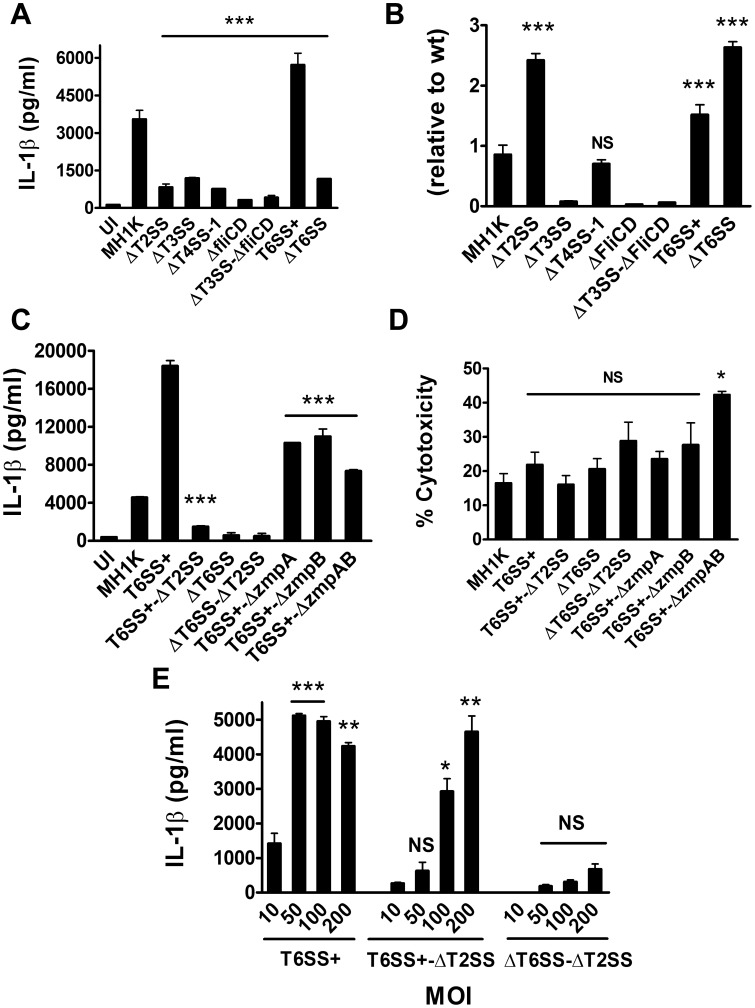
*B. cenocepacia-*induced macrophage IL-1β secretion through cooperation between the T6SS and the T2SS. Graphs represent mean ± SEM from three independent experiments. A. Macrophages were infected with *B. cenocepacia*-MH1K, Δ*T2SS*, Δ*T3SS*, *T4SS-1*, Δ*fliCD*, Δ*T3SS-*Δ*fliCD*, *T6SS*+ or Δ*T6SS* at an MOI of 50 for 1 h and extracellular bacteria removed with gentamicin. ELISA was used to quantify IL-1β in cell supernatants at 24 h post-infection. ***, *p*≤0.001 compared to macrophages infected with MH1K. B. Macrophages were infected as in (A). At 1 h post-infection, the cells were lysed and CFUs were determined. The Invasion Index was calculated relative to MH1K. ***, *p*≤0.001 values compared to macrophages infected with MH1K; NS, non significant. C. Macrophages were infected as in (A) with *B. cenocepacia* MH1K, *T6SS*+, *T6SS*+-Δ*T2SS*, Δ*T6SS*, Δ*T6SS-*Δ*T2SS*, *T6SS*+-Δ*zmpA*, *T6SS*+-Δ*zmpB* or *T6SS*+-Δ*zmpAB*. ELISA was used to quantify IL-1β in cell supernatants at 24 h post-infection. ***, *p*≤0.001 values compared to macrophages infected with T6SS+. D. Macrophages were infected with the same bacterial strains as in (C). Supernatants of infected macrophages were used to quantify the total LDH activity at 24 h post-infection. *, *p*≤0.05 values compared to macrophages infected with MH1K; NS, non significant. E. Macrophages were infected with *T6SS+*, *T6SS+-*Δ*T2SS* and Δ*T6SS-*Δ*T2SS* at different MOI for 1 h and then treated with gentamicin to remove extracellular bacteria. ELISA was used to quantify IL-1β in cell supernatants at 24 h post-infection. *, *p*≤0.05; **, *p*≤0.01 and ***, *p*≤0.001 values compared to macrophages infected with *B. cenocepacia* mutants at an MOI of 10. NS, non significant.

Specialized secretion systems of Gram-negative intracellular pathogens deploy bacterial proteins into eukaryotic cells with the ability to alter phagocytosis and phagosomal maturation, as well as inducing or preventing host cell death [32], [33]. *B. cenocepacia* has two T4SS (the plasmid-encoded T4SS-1 and chromosome 2-encoded T4SS-2) [34], one T3SS, and one T6SS. To investigate whether induction of IL-1β secretion by *B. cenocepacia-*infected macrophages depends on a specific secretion system, we performed infections with mutants carrying partial or complete deletions in the genes encoding each of these secretion systems that render them nonfunctional (Table 1). We also included a deletion mutant defective in T2SS, a secretory pathway that is required for secretion of toxins, proteases, cellulases, and lipases across the bacterial outer membrane [35], [36]. Δ*fliCD*, a mutant unable to produce flagellin, was also used to rule out IL-1β secretion by released flagellin into the macrophage cytosol [37]. Infections of macrophages with Δ*T2SS*, Δ*T3SS*, Δ*T4SS-1*, and Δ*T6SS* mutants were associated with reduced IL-1β secretion (Figure 4A). In contrast, macrophages infected with Δ*atsR* (herein *T6SS+*), lacking a negative regulator that results in the overexpression of the T6SS [24], showed higher levels of IL-1β secretion than those infected with the parental MH1K, while infection with Δ*T4SS-2* does not affect IL-1β secretion (data not shown).

To demonstrate whether reduced IL-1β secretion is directly associated with the absence of the bacterial secretion system assessed and not to decreased bacterial infectivity, we quantified the CFUs at 1 h post-infection and determined the invasion index relative to the parental (*wt*) strain. Δ*T3SS*, Δ*fliCD* and Δ*T3SS-*Δ*fliCD* had decreased ability to infect macrophages, suggesting that the reduced levels in IL-1β secretion were associated with reduced bacterial load inside macrophages. In contrast, Δ*T2SS*, Δ*T4SS-1*, Δ*T6SS*, and *T6SS*+ had equal or better infectivity than the parental strain (Figure 4B). These results suggest that the T2SS, T4SS-1, and T6SS contribute directly to IL-1β secretion by infected macrophages. While T6SS and T4SS-1 can deliver effectors across the eukaryotic cell membrane and the membrane of bacteria-containing vacuoles [25], [34], [38], the T2SS can only mediate secretion of proteins to the bacterial extracellular milieu but not directly into the host cell cytoplasm. This suggested the possibility that a cooperation exists between the T6SS or T4SS-1 and the T2SS to induce IL-1β secretion.

Since Δ*T2SS* and Δ*T6SS* showed almost identical high-level infectivity in macrophages compared to MH1K and Δ*T4SS-1*, we first investigated whether T2SS and T6SS might function synergistically. The T2SS secretes ZmpA and ZmpB proteases, which proteolytically cleave various substrates including cytokines [27], [39], [40]. Therefore, to test our hypothesis we constructed a set of isogenic deletion mutants in the T2SS and T6SS gene clusters, as well as in z*mpA* and *zmpB*. Macrophages infected with *T6SS*+-Δ*T2SS*, Δ*T6SS*, and Δ*T6SS-*Δ*T2SS* failed to produce high amounts of IL-1β (Figure 4C). Also, infected macrophages with *T6SS*+-Δz*mpA*, *T6SS*+-Δ*zmpB* and *T6SS*+-Δ*zmpAB* displayed 50% reduction in IL-1β secretion relative to macrophages infected with *TSS6*+. Since none of these mutants differ in invasion index (data not shown), we conclude that ZmpA and ZmpB contribute directly to IL-1β secretion (Figure 4C). The differences observed in IL-1β secretion by macrophages infected with these mutant strains and Δ*T2SS* suggest that other T2SS substrates may also contribute to the release of IL-1β. In all cases, macrophage infection with these mutants did not increase cytotoxicity (Figure 4D). To validate that decreased IL-1β secretion is due to the lack of T2SS and T6SS function and not to a defect in macrophage invasion, we infected macrophages with *T6SS*+-Δ*T2SS* and Δ*T6SS-*Δ*T2SS* at different MOIs (10, 50 100 and 200). Both mutants failed to induce IL-1β secretion compared to *T6SS*+ at MOIs of 10 and 50 (Figure 4E). At higher MOIs (100 and 200), only Δ*T6SS-*Δ*T2SS* failed to induce IL-1β secretion. *T6SS*+-Δ*T2SS*, *T6SS*+-Δ*zmpA*, *T6SS*+-Δ*zmpB*, and *T6SS*+-Δ*zmpAB* retain the ability to induce disruption of the actin cytoskeleton (Figure S2), a property associated with a functional T6SS [9], [24], indicating that the absence of a functional T2SS and its substrates ZmpA and ZmpB does not compromise T6SS function. Together, these experiments suggest that the T2SS effector proteins depend on a functional T6SS to mediate IL-1β secretion.

### The T2SS and its Secreted Proteins ZmpA and ZmpB are Required for *B. cenocepacia* Intramacrophage Survival

**Figure 5:**
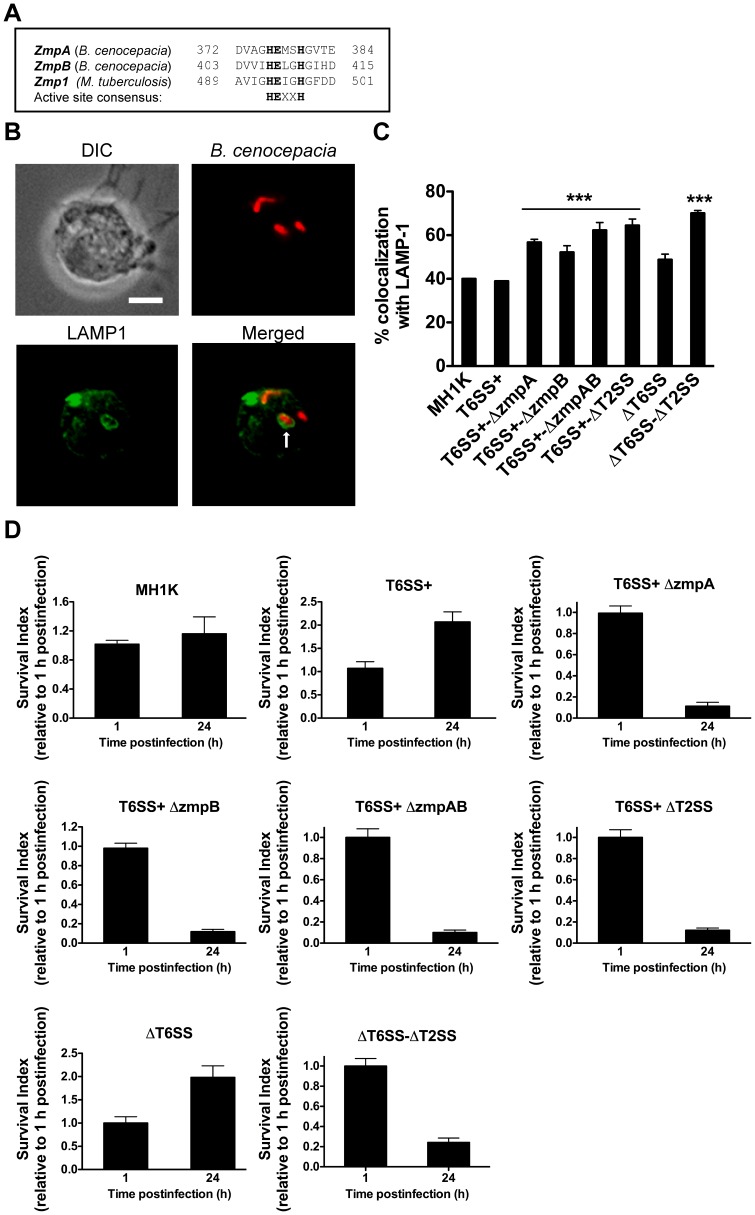
T2SS and its secreted substrates ZmpA and ZmpB are required to delay phagolysosomal fusion and for bacterial survival in macrophages in a T6SS-independent manner. A. Alignment of the predicted ZmpA and ZmpB from *B. cenocepacia* with Zmp1 from *Mycobacterium tuberculosis*. The active site consensus is indicated in bold. B. Macrophages were infected with *B. cenocepacia* MH1K*-*RFP (Red) at an MOI of 50 for 4 h. Infected cells were fixed, permeabilized and stained with anti-LAMP-1 antibodies (green). Stained cells were analyzed by immunofluorescence microscopy. The arrow indicates LAMP-1 associated with the BcCV. Bar represents 10 µm. C. Macrophages were infected with *B. cenocepacia* MH1K, *T6SS+*, *T6SS+*-Δ*zmpA*, *T6SS+*-Δ*zmpB, T6SS+*-Δ*zmpAB, T6SS+*Δ*T2SS*, Δ*T6SS* or Δ*T6SS*-Δ*T2SS,* all expressing the red fluorescent protein (Red), for 1 h. Extracellular bacteria were removed by gentamicin treatment. Infected cells were fixed, permeabilized and stained with anti-LAMP1 antibodies (green). Quantification of LAMP1 associated with the BcCV is shown. Graph represents mean ± SEM from independent experiments including at least 60 vacuoles per experiment. *** *p*≤0.001 relative to LAMP1 associated to MH1K. (D) Macrophages were infected as in (C) and lysed at 1 and 24 h post-infection to quantify CFUs. The survival index at 24 h was calculated relative to the number of CFUs at 1 h post-infection. Graphs represent mean ± SEM from three independent experiments.

Macrophages infected with the T6SS mutants do not have defects in cell migration and in in the assembly of NADPH oxidase onto the BcCV membrane, but the maturation of the BcCV continues to be arrested [9]. Moreover, mutants defective in the T6SS [24], [41], as well as in ZmpA and ZmpB production [26], [39] are attenuated *in vivo*. ZmpA and ZmpB contain the conserved, active site consensus sequence HEXXH (Figure 5A). This sequence is also present in the Zmp1 metalloprotease from *Mycobacterium bovis,* which mediates inhibition of lysosome fusion and inflammasome activation [42]. Therefore, we utilized our set of mutants to investigate whether ZmpA and ZmpB could confer on *B. cenocepacia* the ability to delay phagolysosomal fusion. Macrophages infected with the parental strain showed that 40±0.2% of BcCVs acquire LAMP-1 by 4 h post-infection (Figure 5B and 5C), as we have previously demonstrated [6], [9]. In contrast, 64±3% of BcCVs in macrophages infected with Δ*T2SS* acquired LAMP-1. Similar results were found in macrophages infected with *TSS6*+-Δ*zmpA* (57±3%), *T6SS*+-Δ*zmpB* (53±3%) and *T6SS*+-Δ*zmpAB* (62±4%) mutants, but not with those infected with Δ*T6SS* (48±2%) (Figure 5C). Increased LAMP-1 colocalization with BcCVs containing *T6SS*+-Δ*T2SS*, *T6SS*+-Δ*zmpA*, *T6SS*+-Δ*zmpB*, and *T6SS*+-Δ*zmpAB* suggests that the T2SS and its effector proteins are important to delay phagolysosomal fusion independently of the T6SS. To assess if the T2SS, ZmpA and ZmpB are also required for intramacrophage bacterial survival, we determined the CFUs of infected macrophages at 1 h and 24 h post-infection using a gentamicin protection assay. For these experiments, we utilized gentamicin-sensitive mutants derived from the *B. cenocepacia* MH1K, which is highly sensitive to gentamicin but shows identical trafficking in macrophages to the parental strain K56-2 [28]. As expected, *T6SS*+ and Δ*T6SS* survived and replicated in macrophages (Figure 5D), whereas *T6SS*+-Δ*T2SS*, *T6SS*+-Δ*zmpA*, *T6SS*+-Δ*zmpB*, *T6SS*+-Δ*zmpAB*, and Δ*T6SS-*Δ*T2SS* mutants had significantly reduced survival (Figure 5D). Therefore, the T2SS and its substrates ZmpA and ZmpB contribute to the BcCV maturation defect and to bacterial survival in macrophages.

### A Functional T6SS is Required for the Translocation of T2SS Secreted Proteins to the Macrophage Cytoplasm

**Figure 6:**
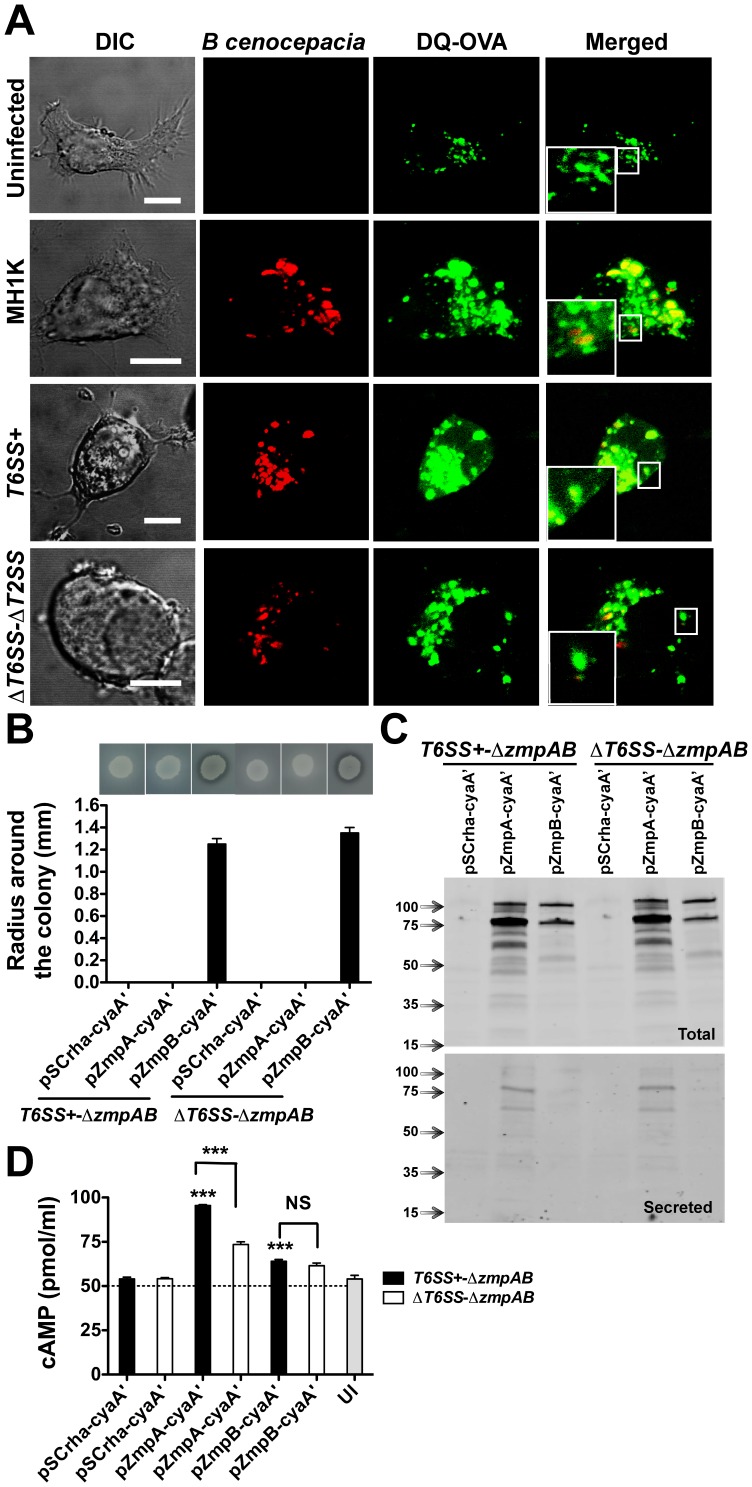
BcCV membrane disruption and translocation of T2SS-secreted proteins ZmpA and ZmpB into the cytosol of infected macrophages. A. Preloaded macrophages with DQ-OVA (Green) were infected with *B. cenocepacia* MH1K-, *T6SS+*- and Δ*T6SS*-Δ*T2SS*-RFP (Red) for 6 h. The cells were analyzed by confocal microscopy. Insets are higher magnifications of the boxed areas. Bars represent 10 µm. B. *B. cenocepacia T6SS+*-Δ*zmpAB* and Δ*T6SS-*Δ*zmpAB* were transformed with pSCrha-cyaA’, pZmpA-cyaA’ and pZmpB-cyaA’. The culture used to infect macrophages was induced with 0.2% of rhamnose for 1 h to allow expression of the protein fusion under the control of the rhamnose-inducible promoter. The functionality of the fusion proteins was tested for proteolysis of casein-D-BHI agar-plates at 37°C over 24 h. Image at the top of the graph shows the radius of proteolysis around the colony. Graph represents mean ± SEM from two independent experiments. C. *B. cenocepacia T6SS+*-Δ*zmpAB* and Δ*T6SS-*Δ*zmpAB* were transformed with pSCrha-cyaA’, pZmpA-cyaA’ and pZmpB-cyaA’ and grown in 25 ml of LB medium in the presence of 0.02% rhamnose for 3 h. The supernatants were precipitated with TCA and the proteins were resolved in SDS-PAGE and analyzed by western blotting. Blots were probed with anti-cyaA’ antibodies. D. Macrophages were infected for 4 h with induced *B. cenocepacia T6SS+*-Δ*zmpAB* and Δ*T6SS-*Δ*zmpAB* transformed with pSCrha-cyaA’, pZmpA-cyaA’ and pZmpB-cyaA’ plasmids. Infected cells were lysed to measure cAMP generation. *** *p*≤0.001, values compared to macrophages infected with *T6SS+*-Δ*zmpAB*(pCyaA’). NS, non-significant.

T6SS has been proposed to have the ability to puncture the phagosomal membrane [25]. The requirement for both the T6SS and the T2SS to induce IL-1β secretion (Figure 4) suggests that the T6SS could mediate phagosomal membrane disruption resulting in the release of T2SS-secreted proteins from the BcCV into the host cytoplasm. To investigate this possibility, macrophages were preloaded with DQ-OVA (green), and infected with *B. cenocepacia* MH1K, *T6SS*+, and Δ*T6SS*-Δ*T2SS* expressing RFP (Red). At 6 h post-infection, uninfected macrophages and macrophages infected with the Δ*T6SS*-Δ*T2SS* mutant had DQ-OVA fluorescence predominantly localized in vacuoles (Figure 6A). In contrast, 61% of the fluorescence in macrophages infected with MH1K and 71% of the fluorescence in macrophages infected with *T6SS*+ appeared not only in the BcCVs but was also dispersed throughout the cell cytoplasm (Figure 6A, insets), suggesting that a functional T6SS is associated with disruption of the integrity of the BcCV membrane.

To investigate whether T2SS-secreted ZmpA and ZmpB could escape from the BcCV we constructed chimeric proteins fused to the 43-kDa, calmodulin-activated adenylate cyclase from *Bordetella pertussis* [43], which is only functional in the host cytosol. We first examined whether the CyaA fusion affects proteolytic activity and/or the secretion of ZmpA and ZmpB. Since ZmpB, but not ZmpA, shows proteolytic activity on casein [39], only the proteolytic activity of the ZmpB-cyaA’ fusion protein was detected in casein-agar plates (Figure 6B). However, both ZmpA-cyaA’ and ZmpB-cyaA’ fusions were secreted, and that ZmpA-cyaA’ was better expressed that ZmpB-cyaA’, as detected by western blotting (Figure 6C). ZmpA-cyaA’ and ZmpB-cyaA’ are expressed as pre-proenzyme precursors (105- and 106-kDa, respectively, of which the catalytic domain of cyaA’ contributes 43-kDa) that are autocatalytically cleaved into mature ZmpA-cyaA’ or ZmpB-cyaA’ (79- and 78-kDa, respectively) and degradation products. Mature form and degradation products were detected in culture supernatants (Figure 6C). In addition, both mutants show similar growth rates suggesting that the expression of ZmpA-cyaA’ and ZmpB-cyaA’ does not affect bacterial viability (data not shown). Macrophages were infected with *T6SS*+-Δ*zmpAB* and Δ*T6SS-*Δ*zmpAB* carrying the plasmids pZmpA-cyaA’ and pZmpB-cyaA’, and cell free extracts assessed for cAMP production. Uninfected macrophages produced 55±2 pmol/ml of cAMP, while infected macrophages with mutants carrying the plasmid vector produced 54±1 pmol/ml, indicating that bacterial infection alone does not alter the basal levels of endogenous cAMP. In contrast, macrophages infected with *T6SS*+-Δ*zmpAB* carrying pZmpA-cyaA’ produced 95.5±0.5 pmol/ml of cAMP (*p*<0.001; Figure 6D). We conclude that the increased cAMP level depends on the T6SS-dependent release of ZmpA-cyaA’ into the cytosol. However, macrophages infected with Δ*T6SS-*Δ*zmpAB*(pZmpA-cyaA’) also display cAMP levels higher than the basal levels of uninfected macrophages (73.5±1.5 pmol/ml), suggesting an additional mechanism to translocate ZmpA independently of the T6SS. Lysates of macrophages infected with *T6SS*+-Δ*zmpAB*(pZmpB-cyaA’) and Δ*T6SS-*Δ*zmpAB*(pZmpB-cyaA’) both showed higher levels of cAMP (64.0±1.0 and 61.5±1.5 pmol/ml, respectively; Figure 6D) compared to controls, but no different regardless of the functionality of the T6SS, suggesting that ZmpB may be released by a T6SS-independent mechanism. Together, the results of these experiments indicate that ZmpA and ZmpB can be released from the BcCV into the macrophage cytosol by T6SS-dependent and -independent mechanisms, suggesting a disruption of the BcCV membrane during infection by *B. cenocepacia*.

## Discussion

We have recently reported that the intracellular infection of macrophages with *B. cenocepacia* K56-2 is associated with caspase-1-dependent release of IL-1β [16]. In this work, we confirm these results and demonstrate that IL-1β release is also accompanied by activation of the NLRP3 inflammasome, and pyroptosis. We also provide evidence indicating increased damage to the plasma membrane of infected macrophages. Although not shown directly in our work, the presence of the LAMP1 phagolysosomal marker on the cell surface strongly suggests repair of membrane damage by direct fusion of secretory lysosomes with the cell membrane. This is also suggested by the release of lysosomal content into the medium, as indicated by the increased β-galactosidase activity upon infection of macrophages with *B. cenocepacia,* which agrees with a recent report showing that IL-1β secretion is accompanied by lysosome exocytosis [44].

One mechanism for NLRP3 activation involves the secretion of pore-forming bacterial toxins [17], [45]. Pore-forming bacterial toxins usually require lysosomal fusion, and activate upon decrease of phagolysosomal pH [46]. This is not the case for intracellular *B. cenocepacia,* which survive in a maturation-arrested phagosome. Indeed, the BcCV shows delayed acquisition of cytosolic subunits of the vacuolar ATPase and the NADPH oxidase [9], delayed acidification [6], and inactivation of Rab7 [7]. Therefore, a pore-forming toxin cannot explain the mechanism of caspase-1 activation and NLRP3 engagement in *B. cenocepacia*-infected macrophages. Another proposed mechanism for caspase-1 activation is the release of cathepsin B by membrane disruption of lysosomes. Because BcCVs show a marked delay in phagolysosomal fusion and retain a high pH for several hours post-infection [6], we speculate that cathepsin B plays at most a minimal role in NLRP3 inflammasome activation subsequent to *B. cenocepacia* infection.

Bacteria may introduce toxins into host cells by specialized secretion systems such as the T3SS, T4SS and T6SS, which can mediate direct translocation of effector proteins into the host cytoplasm [32], [33]. Of these, the T6SS shares structural homology with the T4 bacteriophage tail-spike [47], suggesting that this secretory system can puncture target cell membranes to translocate effector proteins [25]. We have recently documented that a functional *B. cenocepacia* T6SS plays no direct role in the arrested maturation of the BcCV [9]. If the T6SS apparatus can indeed puncture the phagosomal membrane as proposed, it would be conceivable to expect that cellular components trapped into the BcCVs escape into the host cell cytosol in a T6SS-dependent fashion, as suggested by the dispersion of DQ-OVA fluorescence from the BcCV into the cytoplasm. One system involved in the secretion of bacterial enzymes and toxins is the T2SS [48]. The T2SS, also known as the general secretion pathway, typically handles large proteins that are secreted into the periplasmic space where they fold and are then exported across the bacterial outer membrane into the external milieu [48]. Here, we provide evidence that suggests cooperation between the T2SS and T6SS, resulting in the release of T2SS-secreted proteins into the macrophage cytosol. We show that a functional T6SS is required for the translocation of ZmpA, a T2SS-secreted metalloprotease, into the macrophage cytoplasm, as demonstrated by production of cAMP mediated by a ZmpA-cyaA’ fusion protein. We could not demonstrate a similar phenotype for ZmpB, another T2SS-secreted protease. However, both ZmpA and ZmpB were required for increased IL-1β secretion, suggesting that ZmpB may also reach the cytosol, but perhaps in lower amounts than are detectable with the adenylyl cyclase reporter system. The mechanism of increased IL-1β secretion by ZmpA or ZmpB is unknown but the Zmp1 metalloprotease from *M. tuberculosis* has been reported to inhibit inflammasome activation [42]. ZmpA and ZmpB can process a wide range of substrates including host proteins and cytokines [26], [27]; it is therefore conceivable that these metalloproteases can cleave caspase-1 directly or via the activation of a host protease that in turns processes caspase-1. Attempts to show caspase-1 cleavage mediated by ZmpA or ZmpB did not yield any conclusive results (data not shown).

Our experiments also revealed that ZmpA and ZmpB are required for the maturation arrest of the BcCV, and for *B. cenocepacia* survival in macrophages, both of which are T6SS-independent phenomena. Therefore, these proteases may act as virulence factors within the BcCV. Possible mechanisms may involve the cleavage of one or more substrates at the BcCV membrane, which results in the inhibition of the phagolysosomal fusion or alternatively, protect the bacteria from bacteriolytic molecules within the vacuole. It has been shown that ZmpA and ZmpB play a role in bacterial resistance to antimicrobial peptides [27], suggesting this may explain the reduced bacterial survival of the ZmpA- and ZmpB-defective mutants in the BcCV. In addition to ZmpA and ZmpB, it is possible that other T2SS-secreted proteins escape the BcCV and mediate IL-1β secretion. This can be surmised from the data using a deletion mutant impaired in T2SS, which shows a much more dramatic reduction in IL-1β secretion relative to that of the double *zmpAB* deletion mutant. The T2SS secretome characterization, currently underway, will identify additional effectors that may alter host cell physiology upon escape from the BcCV to the cytosol. Also, our present model does not exclude the cooperation of T2SS-secreted products and other secretions systems such as, for example, the T4SS. *B. cenocepacia* has a chromosome 2-encoded (T4SS-2) and plasmid-encoded (T4SS-1) T4SSs, of which only the latter has been associated to bacterial survival in macrophages [38]. Our results showing that the T4SS-1-defective mutant also fails to induce IL-1β secretion suggest that T4SS-1 specific effectors could cooperate with ZmpA, ZmpB or other T2SS secreted proteins to activate the inflammasome. In conclusion, our data reveal an unanticipated role for T2SS in the intracellular survival of an opportunistic pathogen and describe for the first time a novel mechanism for inflammasome activation that involves the cooperation between two bacterial secretory pathways, resulting in pyroptosis via the NLRP3/ASC inflammasome. Whether elicitation of pyroptosis accelerates the clearance of the intracellular bacteria [49] or potentiates the proinflammatory nature of the *B. cenocepacia* infection in susceptible patients awaits future investigation.

## Materials and Methods

### Reagents

Dulbecco's modified Eagle medium (DMEM), DMEM without phenol red, phosphate saline buffer (PBS) and fetal bovine serum (FBS) were from Wisent (St-Bruno, Quebec, Canada). Monoclonal rat anti-LAMP-1 (ID4B) and rabbit anti-cyaA (3D1) were from BD-Biosciences and Santa Cruz Biotechnology, respectively. Anti-56-2 rabbit polyclonal antibody was prepared by immunization with formalin-fixed K56-2. The secondary antibodies Alexa Fluor (AF) 488-conjugated goat anti-rat, AF-680-conjugated goat anti-mouse, 10-kDa AF-488-conjugated dextran, AF-488-conjugated phalloidin, and AF-488-conjugated Annexin V were from Invitrogen. Goat anti-rabbit conjugated to RyDye-800 was from Rockland. Lact-C2-GFP [50] and PLCδ-PH-GFP [51] were provided by S. Grinstein (SickKids Hospital, Toronto, Canada) and T. Balla (National Institute of Child Health and Human Development, National Institutes of Health, Bethesda, USA), respectively.

### Bacterial Strains, Macrophages and Cell Culture Conditions

Strains and plasmids are described in Table 1. Bacteria grew in Luria-Bertani (LB) broth at 37°C with shaking. Plasmid pDSRedT3, expressing Red Fluorescent Protein (RFP) was mobilized into *B. cenocepacia* strains by triparental mating [52]. *B. cenocepacia* strains containing pDSRedT3 were cultured in LB-chloramphenicol (90 µg/ml final concentration). The C57BL/6 murine bone marrow-derived macrophages cell line ANA-1 was obtained from D. Radzioch, McGill University, Montreal, Quebec, Canada [53], and maintained in DMEM-10% FBS and 100 µg/ml Primocin (Invivogen). Macrophages were transfected with pLact-C2-GFP and pPLCδ-PH-GFP using FUGENE reagent (Roche) according to the manufacturer’s instructions. Bone marrow-derived macrophages were isolated from wild-type C57BL/6, Caspase-1^−/−^, Ipaf-1^−/−^, and ASC^−/−^ mice as described elsewhere [16]. Femurs of NLRP3^−/−^ mice were obtained from K. Thirumala-Devi (St Jude Children’s Hospital, Memphis TN, USA). All animal experiments required to obtain bone marrow-derived macrophages were performed according to standard operating protocols approved by the Animal Care Use Committee of the Ohio State University College of Medicine and the St. Jude Children’s Hospital.

### Macrophage Infection Assays

For infection assays, one ml-bacterial aliquots obtained from overnight cultures in LB at 37°C with shaking were washed twice with PBS and resuspended in DMEM plus 10% FBS. Four x 10^5^ macrophages were seeded on coverslips in six- or twelve-well tissue culture plates. Bacteria were added to macrophages at multiplicity of infection (MOI) of 10, 50 or 100, as appropriate. Plates were centrifuged for 1 min at 1 200 RPM and incubated at 37°C under 5% CO_2_ and 95% humidity. Infected macrophages were washed three times with PBS before fixing with 4% paraformaldehyde for 30 min at room temperature (RT). For LAMP-1 detection at the cell surface, infected macrophage cells were blocked with 2% bovine serum albumin, 3% FBS for 1 h, washed twice with PBS prior to incubation with anti-LAMP-1 (clone ID4B) in blocking solution for 1 h at RT, followed by 45 min incubation with the secondary antibody at RT in blocking buffer. Coverslips were mounted on glass slides using fluorescent mounting medium (DAKO Cytomation). Confocal microscopy was performed using a confocor Zeiss LSM 510 laser scanning confocal microscope. When needed, cell supernatants were collected, centrifuged at 10 000 RPM for 5 min at 4°C and stored at −20°C prior to measuring IL-1β, LDH, and β-galactosidase activities. A gentamicin protection assay to quantify intracellular bacterial survival was performed as described [28].

### Flow Cytometry

LAMP-1 at the cell surface was quantified at 4 and 24 h post-infection. Infected cells were collected with PBS-EDTA 0.04%, incubated with blocking solution for 45 min on ice, washed twice with PBS and incubated for 45 min with anti-LAMP-1 in blocking solution on ice, washed twice with PBS and incubated for 45 min with AF-488 chicken anti-rat on ice protected from light. Cells were finally washed three times, resuspended in 1% formaldehyde in PBS and analyzed with a BD FACSCalibur flow cytometer (Becton Dickinson) using the WinMDi software (http://facs.scripps.edu/software.html). For the TUNNEL and Annexin V assays, cells were processed as above. DNA strand breaks were labeled using the *In Situ* Cell Death Detection Kit as directed by the manufacturer’s instructions (Roche Applied Science). To detect phosphatidylserine on the cell surface, infected cells were labeled with Annexin V-AF488 following the manufacturer’s instructions (Invitrogen). Cells were analyzed by flow cytometry as described above.

### Lactate Dehydrogenase, IL-1β, and β-galactosidase Quantification

Macrophages were infected and cultured as described above for the gentamicin protection assay. Supernatants of infected macrophages were collected at various times post-infection and evaluated for the presence of the cytoplasmic enzyme LDH using the Cytotox 96 kit (Promega). The percent cytotoxicity was calculated as follow: [(Experimental LDH – Spontaneous LDH)/(Maximum LDH release – Spontaneous LDH)] × 100. The IL-1β concentration in supernatants of infected macrophages was quantified by ELISA (R&D systems) as described [9]. Experiments were carried out in 96 flat-bottom well plates. Fifty µl of supernatants were incubated with 100 µl of 10 mM of p-nitrophenyl-β-D-galactopyranoside (Sigma-Aldrich) in 150 mM citrate buffer (pH 3.5) for 24 h at 37°C. The reaction was stopped by the addition of 150 µl of 0.5 M of NaCO_3._ The absorbance at 405 nm (A_405_) was measured by Perkin Elmer spectrophotometer and the total β-galactosidase (β-gal) activity was calculated as follows: [(Experimental β-gal – Spontaneous β-gal)/(Maximum β-gal – Spontaneous β-gal)] × 100.

### Preparation of Proteins from Bacterial Culture Supernatants and Western Blot

Proteins from culture supernatants were prepared as described [24], with minor modifications. Overnight cultures were diluted to an OD_600_ of 0.005 in 25 ml of pre-warmed LB in the presence of 0.02% rhamnose. After 4-5 h of incubation at 37°C, the cultures were centrifuged for 20 min at 14,000 RPM at 4°C. Culture supernatants were sterilized through a 0.22-µm filter (Millipore), and proteins were precipitated overnight at 4°C with 10% (v/v) of trichloroacetic acid (final concentration). The precipitates were isolated by centrifugation at 20 000 RPM for 30 min. The pellets were air dried and then solubilized by the addition of 0.1 M sodium phosphate buffer, pH 7.0. The protein concentration of each sample was determined by Bradford assay (Bio-Rad), and aliquots of 10 µg were loaded on a 16% SDS-PAGE gel. Proteins were transferred onto nitrocellulose membranes and the membranes were blocked overnight at 4°C in 10% blocking solution (Amersham) in PBS-Tween 20 (0.2%), and incubated with the monoclonal antibody anti-cyaA’ overnight at 4°C in 10% of blocking solution in PBS-Tween 20 (0.2%). The blot was washed with PBS-Tween 20 (0.2%), incubated with the appropriate secondary antibody for 1 h at room temperature in 10% blocking solution in PBS-Tween 20 (0.2%). The secondary antibodies were removed by several washes with PBS-Tween20 (0.2%) prior to analyzing the membranes by infrared imaging using an Odyssey imager (LICOR Odyssey). Densitometry was calculated using ImageJ (http://http://rsbweb.nih.gov/ij/).

### Measurement of Intracellular cAMP

Macrophages (2×10^6^) were infected with *B. cenocepacia*-T6SS+Δ*zmpAB* and ΔT6SSΔ*zmpAB* carrying the following plasmids: pSCrha-cyaA’, pZmpA-cyaA’ and pZmpB-cyaA’ at a MOI of 50 for 6 h. After infection, the cells were washed with cold PBS, lysed quickly and subjected to the cyclic AMP direct immunoassay (Arbor Assays) following the manufacturer’s instructions.

### Detection of Total Proteolytic Activity

Macrophages were seeded onto glass coverslips. The macrophages were pre-loaded with DQ-OVA (Invitrogen) during one hour, then cells were washed twice with PBS and the culture was continued by one hour. Pre-loaded macrophages with DQ-OVA were infected with *B. cenocepacia* MH1K-RFP for 2 or 6 h in the presence of DQ-OVA. Infected cells were washed twice with PBS, fixed and the coverslips were mounted on glass slides using fluorescent mounting medium (DAKO Cytomation). The samples were analyzed using a confocor Zeiss LSM 510 laser scanning confocal microscope. At the same time, pre-loaded macrophages with DQ-OVA were infected with *B. cenocepacia* MH1K during 2 or 4 h. Infected cells were washed twice times with PBS, then lysed with SDS (1% final concentration); the total proteolytic activity was quantified in a spectrofluorometer (Ex _480nm_, Em_480-600nm_).

### General Molecular Techniques

Restriction enzymes, T4 DNA ligase, and alkaline phosphatase were obtained from Roche Diagnostics (Laval, Quebec, Canada). DNA transformations of *E. coli* DH5α and *E. coli* SY327 were performed by the calcium chloride method [54]. Conjugations into *B. cenocepacia* were performed by triparental mating [52] using the helper pRK2013 [55]. DNA amplifications by PCR were done with the PTC-0200 or PTC-221 DNA engine (MJ Research, Incline Village, NV) with HotStar HiFidelity DNA polymerase (Qiagen). DNA sequencing was performed at the DNA sequencing facility of York University (Toronto, Canada). The computer program BLAST was used to analyze the sequenced genome of *B. cenocepacia* strain J2315.

### Mutagenesis of *B. cenocepacia* and Complementing Plasmids

Unmarked and non-polar gene deletions were performed as described [56]. Details about the construction of the deletion plasmids will be published elsewhere. All gene deletions in *B. cenocepacia* were confirmed by PCR. The calmodulin-activated adenylate cyclase domain from the *Bordetella pertussis* cyclolysin (*cyaA’*) was PCR amplified using primer pairs 3123/3138 (5′-TTTTAAGCTTTGTCATAGCCGGAAT/5′-ATATTCTAGACAGCAATCGCATCAGG) and pMS107 as a template [43].

The amplicons were digested with *Hin*dIII-*Xba*I and cloned into similarly digested pSCrhaB2 [57], giving rise to pSCrha-cyaA’, a rhamnose inducible plasmid allowing for the creation of CyaA’ fusion proteins. *zmpA* and *zmpB* were amplified using primer pairs 5647/5648 (5′-AAACATATGACAAACCCATTCATAACCT/5′- AAAATCTAGAATTCACCCCGACCGCACT) and 5649/5650 (5′-AAAACATATGCGTGTCCAACCGTTGAGATG’/AAAATCTAGACGACTTCTGCGGGACGGTCA), respectively. PCR products were digested with *Nde*I-*Xba*I and cloned into similarly digested pSCrha-cyaA’, resulting in pZmpA-cyaA’ and pZmpB-cyaA’ encoding ZmpA and ZmpB proteins C-terminally fused to the CyaA’ domain, respectively. The expression of CyaA’ fusion proteins was induced in bacterial cultures by the addition of rhamnose (0.2% w/v).

### Statistical Analysis

Statistical analyses were done by two-way ANOVA and Student’s *t*-test, as appropriate, using GraphPad Prism version 4.03.

## Supporting Information

Figure S1 **Intracellular proteolytic activity of macrophages infected with** ***B. cenocepacia*** **.** A. Macrophages were infected with *B. cenocepacia* MH1K-RFP (Red) for 2 and 6 h in the presence of DQ-OVA (Green) and analyzed by confocal microscopy. Bar, 10 µm. B. Macrophages were infected with *B. cenocepacia* MH1K and *T6SS+* as in (A). Infected cells were washed twice and lysed with SDS (1% final concentration); the total intracellular proteolytic activity was quantified in a spectrofluorometer (Ex _480nm_, Em_480–600nm_). Graph represents mean ± SEM of three independent experiments.
(TIF)

Figure S2 **Loss of functionality in the T2SS, ZmpA, and ZmpB does not impair the ability of intracellular** ***B. cenocepacia*** **to induce disruption of the actin cytoskeleton.** Macrophages were infected with *B. cenocepacia* MH1K, *T6SS+*, *T6SS+*Δ*zmpA*, *T6SS+*Δ*zmpB, T6SS+*Δ*zmpAB, T6SS+*Δ*T2SS*, Δ*T6SS* or Δ*T6SS*Δ*T2SS* for 4 h. The cells were fixed and analyzed by light microscopy. Bar, 30 µm.
(TIF)
