# Supplementary figures and images for: Burkholderia cenocepacia Type VI Secretion System Mediates Escape of Type II Secreted Proteins into the Cytoplasm of Infected Macrophages
Source: PLoS One. 2012 Jul 25;7(7):e41726. doi: 10.1371/journal.pone.0041726 (PMC3405007; doi:10.1371/journal.pone.0041726)

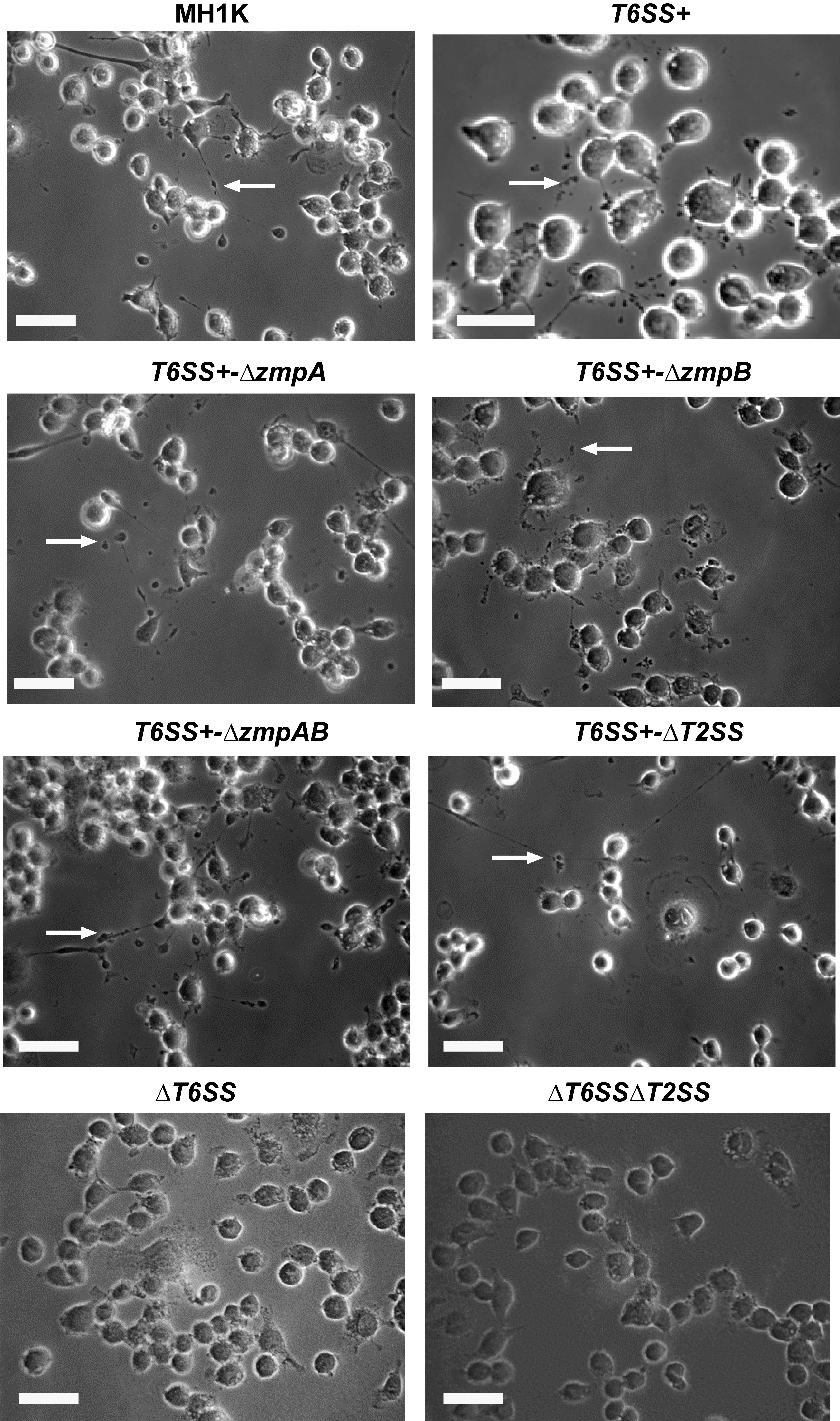

Supplement: Figure S2 — Loss of functionality in the T2SS, ZmpA, and ZmpB does not impair the ability of intracellular B. cenocepacia to induce disruption of the actin cytoskeleton. Macrophages were infected with B. cenocepacia MH1K, T6SS+, T6SS+ΔzmpA, T6SS+ΔzmpB, T6SS+ΔzmpAB, T6SS+ΔT2SS, ΔT6SS or ΔT6SSΔT2SS for 4 h. The cells were fixed and analyzed by light microscopy. Bar, 30 µm. (TIF) [file pone.0041726.s002.tif]
